# Comment on “Antibiotic Resistance Profiles of *Haemophilus influenzae* Isolates from Children in 2016: A Multicenter Study in China”

**DOI:** 10.1155/2021/5096801

**Published:** 2021-02-17

**Authors:** Meltem Karabay, Oguz Karabay

**Affiliations:** ^1^Sakarya University Faculty of Medicine, Department of Neonatology, Sakarya, Turkey; ^2^Sakarya University Faculty of Medicine, Department of Infectious Diseases, Sakarya, Turkey

We read with great interest the article by Wang et al. recently published in *BioMed Research International*. [1] The authors identified antibiotic susceptibility of *Haemophilus influenzae* strains obtained from six children's hospitals in different regions of China. However, we have two concerns with the study. Upon careful examination of the distribution of the obtained samples, we understood that 1,558 (75%) samples were obtained from sputum and 107 (5%) samples were obtained from throat swabs. Firstly, it is necessary to mention that the presence of *H. influenzae* in the throat specimens or sputum does not indicate that it is a causative agent. *H. influenzae* is one of the most common contaminants, especially in throat and sputum samples. The presence of this bacterium in sputum or some clinical specimens does not indicate that *H. influenzae* is the cause of the disease. Therefore, the results of the antibiograms obtained from these samples cannot be evaluated as if they were patient samples.

Secondly, we found that *H. influenzae* was isolated in 231 vaginal specimens. Some of the most frequent and expected pathogenic causes of vaginitis are *Candida* spp., *Trichomonas vaginalis*, and *Gardnerella vaginalis*. In contrast, *Haemophilus* spp. are not a commonly expected cause of vaginitis. We are therefore of the opinion that the 231 (11%) vaginal *H. influenzae* strains are likely to be only contamination. As a result, we think that many of the results of this antibiogram obtained by the authors do not show causative bacteria, but rather flora member bacteria indicating the presence of flora rather than the disease. The results may have been more conclusive if the study had aimed to determine the resistance rate of *H. influenzae* and collected data from sterile tissue samples such as blood and CSF.
